# Supplementary material for: The Gompertz Law emerges naturally from the inter-dependencies between sub-components in complex organisms
Source: Sci Rep. 2024 Jan 12;14:1196. doi: 10.1038/s41598-024-51669-5 (PMC10786855; doi:10.1038/s41598-024-51669-5)
Supplement: Supplementary file 1 — Supplementary Information. [file 41598_2024_51669_MOESM1_ESM.pdf]

SUPPLEMENTARY MATERIAL

Supplemental Appendix A: Mean field model for constant failure rate of individual subsystems

If subsystems fail at an average constant rate, C, we have $p(t) = C \cdot \Delta t$ and:

$$\Delta F = C \cdot (N - F(t)) \cdot \Delta t \tag{12}$$

The above leads to the differential equation:

$$\frac{dF(t)}{dt} = \lim_{\Delta t \to 0} \frac{\Delta F}{\Delta t} = C(N - F(t)) \tag{13}$$

Which has the solution:

$$F(t) = N - (N - F_0)e^{-Ct} \tag{14}$$

In this case we obtain the following expression for mortality rate:

$$\mu(t) \propto \frac{F(t)}{N} = 1 - (1 - F_0/N)e^{-Ct} \tag{15}$$

This solution does not increase exponentially with time, and is therefore not in agreement with Gompertzian mortality.

Supplemental Appendix B: Linear noise approximation of the master equation

The approximate solution for the master equation (9) can be obtained by performing Van Kampen's system size expansion[39], where we expand the system around the limit $N \to \infty$.

For this, we first redefine the system's parameters such that the limit $N \to \infty$ is well defined, and define a new rate constant $\tilde{r} = Nr$ to be a parameter independent of N. Then eq. (9) becomes

$$\frac{d}{dt}P(f,t) = \frac{\tilde{r}}{N}(N-f+1)(f-1)P(f-1,t) - \frac{\tilde{r}}{N}(N-f)fP(f,t). \tag{16}$$

The number of the failed subsystems at time t, $f(t)$, is a stochastic variable, that fluctuates over time. We divide $f(t)$ into a deterministic part and a fluctuating part as

$$f(t) = N\phi(t) + \sqrt{N}Z(t) \tag{17}$$

This form comes from the expectation from the central limit theorem, where in the infinite system size ($N \to \infty$), the average behavior of $f(t)$ converges to a deterministic mean-field behaviour:

$$\langle f(t) \rangle = N\phi(t) \tag{18}$$

and the individual deviations from this mean-field behavior (the "noise") scales with $\sqrt{N}$:

$$f(t) - \langle f(t) \rangle = \sqrt{N}Z(t) \tag{19}$$

The distribution of the stochastic variable $Z(t)$ is given by $Q(z,t) = \sqrt{N}P(f,t)$, which we assume will have the following form:

$$Q(z,t) = \frac{1}{\sqrt{2\pi\Xi(t)}} \exp\left(-\frac{z^2}{2\Xi}\right), \tag{20}$$

where $\Xi(t)$ is the time-dependent variance of Z.

Following the procedure in ref.[39], we re-write the master equation (16) in terms of $\phi(t)$, $Z(t)$ and $Q(z,t)$. We then truncate the system size expansion up to the linear order in noise term (Linear Noise Approximation, LNA), to obtain the deterministic mean field equation for $\phi(t)$:

$$\frac{d\phi(t)}{dt} = \tilde{r}(1-\phi(t))\phi(t), \tag{21}$$

The obtained rate equation (21) indeed agrees with the mean field model (3) given $\tilde{r} = rN$ and $\phi = \lim_{N\to\infty} \frac{\langle f \rangle}{N}$. Therefore, given (4), the solution for $\phi(t)$ is a logistic growth curve:

$$\phi(t) = \frac{\phi_0}{\phi_0 + (1-\phi_0)e^{-\tilde{r}t}} \tag{22}$$

where ϕ_0 is a constant determined by the initial condition.
For the time-dependent variance of Z, $\Xi(t)$, we obtain the following equation:

$$\frac{d}{dt}\Xi(t) = 2\tilde{r}(1-2\phi)\Xi(t) + \tilde{r}(1-\phi)\phi. \tag{23}$$

And by using the solution for $\phi(t)$ given by , eq. (22) we have:

$$\frac{d}{dt}\Xi(t) = 2\tilde{r}\frac{-\phi_0 + (1-\phi_0)e^{-\tilde{r}t}}{\phi_0 + (1-\phi_0)e^{-\tilde{r}t}}\Xi(t) + \tilde{r}\frac{\phi_0(1-\phi_0)e^{-\tilde{r}t}}{[\phi_0 + (1-\phi_0)e^{-\tilde{r}t}]^2}. \tag{24}$$

The solution is given by

$$\Xi(t) = \frac{e^{2\tilde{r}t}}{[\phi_0 e^{\tilde{r}t} + (1-\phi_0)]^4}\left[C_0 + \phi_0(1-\phi_0)\left(\phi_0^2 e^{\tilde{r}t} + 2\phi_0(1-\phi_0)\tilde{r}t - (1-\phi_0)^2 e^{-\tilde{r}t}\right)\right], \tag{25}$$

where C_0 is an integration constant, which should be determined by the initial value of the variance. If we set initial condition as $\Xi(0) = 0$ (no variance at time zero), we have

$$\Xi(t) = \frac{e^{2\tilde{r}t}}{[\phi_0 e^{\tilde{r}t} + (1-\phi_0)]^4} \left[\phi_0(1-\phi_0)\left(\phi_0^2(e^{\tilde{r}t}-1) + 2\phi_0(1-\phi_0)\tilde{r}t - (1-\phi_0)^2(e^{-\tilde{r}t}-1)\right)\right]. \tag{26}$$

In the small initial failure fraction ϕ_0 limit at finite time t, this can be expanded as

$$\phi(t) = \phi_0 e^{\tilde{r}t} + O(\phi_0^2), \tag{27}$$

and

$$\Xi(t) = \phi_0 e^{2\tilde{r}t}\left(1 - e^{-\tilde{r}t}\right) + O(\phi_0^2). \tag{28}$$

Therefore, for the early time, the variance is expected to grow exponentially over time. The time dependent variance of the original variable f is given by $\langle (f - N\phi(t))^2 \rangle = N\Xi(t)$.

Supplemental Appendix C: Full numerical solution to $P(f,t)$

In order to compute the full distribution of $P(f,t)$, we derive an expression that may be solved numerically for discrete time. If a system has f failed subsystems at time $t+\Delta t$, the system must have had $k \leq f$ failed subsystems at time t and the number of new subsystem failures ("ΔF") that occur within the time-step Δt, must be exactly $f-k$.

$$P(f,t+\Delta t) = \sum_{k=0}^{f} P(k,t) \cdot Prob(k, \Delta F = f-k) \tag{29}$$

The probability $prob(k, \Delta F = f-k)$, describes the probability that a system with k already failed subsystems experiences $f-k$ new failures within the time-step Δt. The number of subsystems "available for failure" is $N-k$. The probability $Prob(k, \Delta F = f-k)$ is therefore given by the Binomial distribution:

$$B^{N-k}_{(f-k)} = \binom{N-k}{f-k} p^{f-k}(1-p)^{N-f} \tag{30}$$

Which gives the probability that out of $(N-k)$ subsystems, $(f-k)$ will fail and $(N-f)$ will not fail. The risk for each subsystem to fail is given by p. In our case p is proportional the number of subsystems that have already failed: $p = r \cdot k \cdot \Delta t$. We therefore have the following recurrence relation:

$$P(f,t+\Delta t) = \sum_{k=0}^{f} P(k,t) \cdot \binom{N-k}{f-k} (r \cdot k \cdot \Delta t)^{f-k}(1 - r \cdot k \cdot \Delta t)^{N-f} \tag{31}$$

From equation (31) we can re-obtain the master equation (9) by taking the $\Delta t \to 0$ limit, and disregarding terms of higher order of Δt. The equation (31) may be implemented numerically to obtain a full distribution of $P(f,t)$ for a discrete set of time-steps and without assuming $N \to \infty$. In Supplementary Figure 1A, we display the median, the upper and lower quartiles, and the 5th and 95th percentiles of the distribution of $P(f,t)$. We note the the solution to the distribution $P(f,t)$ corresponds to the distribution of individual trajectories in the case where death events are ignored. In Supplementary Figure 1B, we display the median and average of the distribution of $P(f,t)$. The mean field solution is also displayed for comparison. We see that the median and average value of $P(f,t)$ are not exactly the same - for early times, the average value is higher than the median. We also observe that the stochastic model lags slightly compared to the mean field model. For this reason the stochastic model used in the article has been implemented with a slightly higher value of the parameter r, such that the results fir the empirical data better (for the mean field model we used $rn = b$ and for the stochastic model we used $rN = b/0.977$).

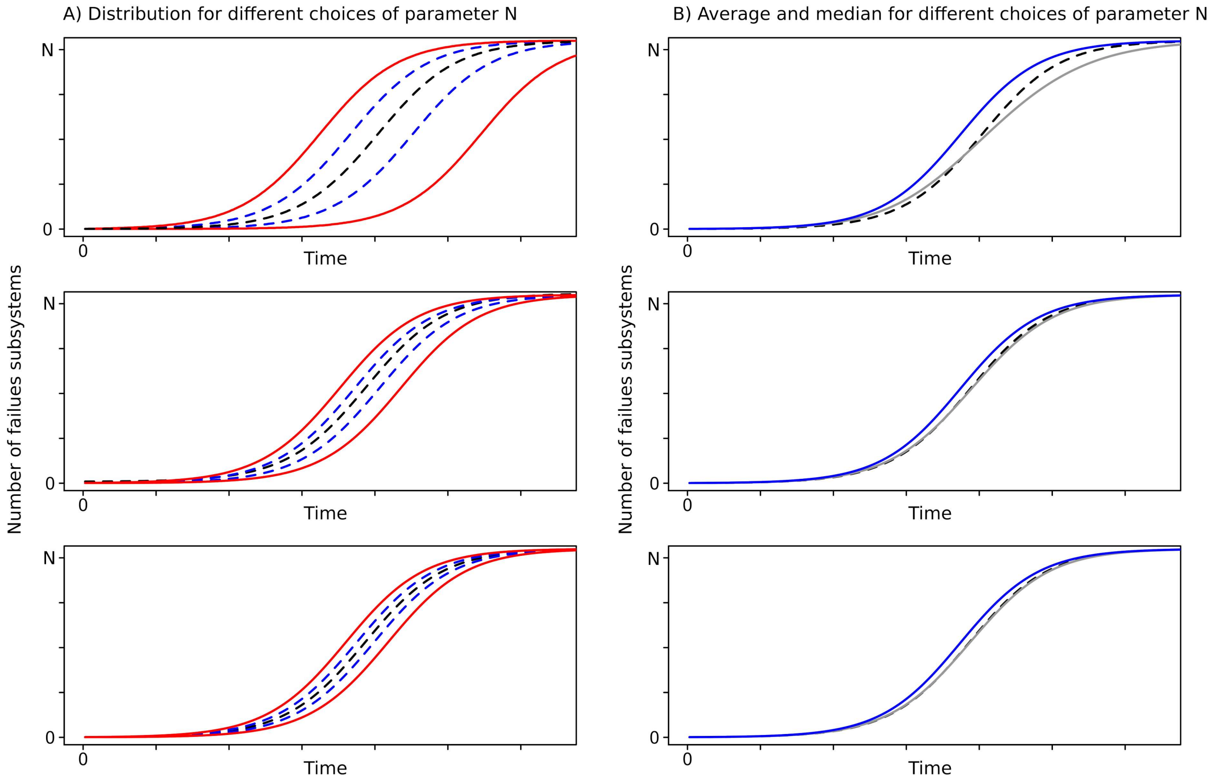

Supplementary Figure 1. A) The distribution of individual trajectories as a function of time, in the case where death events are ignores - i.e. all trajectories are allowed to continue until saturation ($f_i = N$). The black dashed lines represent the median of the distribution, blue dashed lines represent the upper and lower quartiles and full red lines represent the 5th and 95th percentile. The difference between the top, middle and bottom panels is only the choice of f_0/N: Top panel has $f_0/N = 1/1000$, Middle panel has $f_0/N = 2/1000$, Bottom panel has $f_0/N = 5/1000$. B) From the distribution of individual trajectories we show the median (black dashed line - same as in A), the average of the distribution (grey full line) and the corresponding mean field average (blue full line). We see that the mean field model is an increasingly good approximation to the stochastic model when f_0/N increases. The same is true for increasing N, keeping f_0/N constant (data not shown). We also see that for finite the stochastic model displays a small time-lag compared to the mean field model - which is why the implementation of the stochastic model should be fitted to a slightly higher value of b, in order to fit empirical data.
